# Type I collagen promotes tumor progression of integrin β1 positive gastric cancer through a BCL9L/β-catenin signaling pathway

**DOI:** 10.18632/aging.203355

**Published:** 2021-07-28

**Authors:** Yalei Lv, Yujie Shan, Lina Song, Yufei Zhao, Ruixue Lai, Junyan Su, Xiaoyun Zhang

**Affiliations:** 1 Department of Medical Oncology, The Fourth Hospital of Hebei Medical University, Hebei, China; 2 Department of Oncology, Cangzhou Central Hospital, Hebei, China; 3 Department of Immunology and Rheumatology, The Fourth Hospital of Hebei Medical University, Hebei, China; 4 Lifehealthcare Clinical Laboratories, Hangzhou, Zhejiang, China

**Keywords:** type I collagen, integrin β1, BCL9L, β-catenin, gastric cancer

## Abstract

The mechanism of extracellular matrix induced tumor progression is poorly understood. Based on the TCGA database and clinical tumor tissues analysis, we observed abundant type I collagen expression in tumor tissues and poor overall survival in gastric patients with high integrin β1 (ITGB1) expression. *In vitro*, our study found that 3D collagen culture promoted the capability of colony formation and growth in ITGB1 positive gastric cancer, whereas limited colony growth was observed in ITGB1 negative gastric cancer, suggesting the role of ITGB1 in type I collagen associated tumor progression. Mechanistically, we demonstrated that type I collagen was capable of promoting the activation of BCL9L/β-catenin signaling pathway through ITGB1, thereby contributing to the gastric cancer development. Subsequently, β-catenin signals further up-regulated the expression anti-apoptosis protein BCL2, leading to the chemo-resistance in gastric cancer cells. Blockade of β-catenin signals efficiently improved the anticancer effects of chemotherapy, providing an innovative sight for clinical gastric cancer therapy.

## INTRODUCTION

Gastric cancer is one of the most common malignant carcinomas with the third leading causes of cancer-associated death worldwide [1]. Surgery combing adjuvant chemotherapy is usually implemented for gastric cancer patient treatment in early stage [2]. In metastatic disease, gastric cancer patients suffered poor outcome, with a median survival being around 1 year due to the sustained tumor progression and developed chemo-resistance [3]. However, the underlying mechanism of tumor progression in gastric cancer remains controversial, and there is urgent need to explore innovative approach to elevate the treatment efficiency in gastric cancer therapy.

Cancer development is bound up with diverse biological process, including extracellular matrix alterations, which influence the tumor cells proliferation, integrins associated signaling pathways activation and cellular focal adhesion formation [4–6]. Increasing evidence has suggested that extracellular matrix could provide protection against chemotherapy-induced cells apoptosis, and mediate pro-survival signaling pathways activation through bounding to integrins receptors on tumor cells [7, 8]. Various compounds in extracellular matrix have been suggested to be correlated with tumor progression, including type I collagen [9] and laminin [10]. However, the potential role of type I collagen, the major component in extracellular matrix, still remains controversial. Previous investigation has implicated that type I collagen could facilitate tumor stemness and promote the cells metastasis in a 3D collagen gel [11, 12]. However, the statistical analysis based on TCGA database suggested a poor correlation between the type I collagen expression and tumor prognosis in patients. Additionally, the role of matrix components in tumor progression is currently unclear, and the underling mechanism of cancer development induced by extracellular matrix has yet to be developed.

Here, our study further explored the role of type I collagen in gastric cancer based on TCGA database and clinical gastric tumor tissues analysis. We provided evidence that type I collagen could mediated the gastric cancer progression and chemotherapy resistance through an ITGB1 dependent manner. Moreover, we further disclosed the underlying of collagen/ITGB1 induced tumor progression, which was dependent on a BCL9L/β-catenin/BCL2 signaling pathway. Suppression of β-catenin signals efficiently improved the outcome of chemotherapy, which provided an innovative approach for gastric cancer treatment.

## RESULTS

### ITGB1 expression promoted tumor progression in gastric cancer

**Figure 1:**
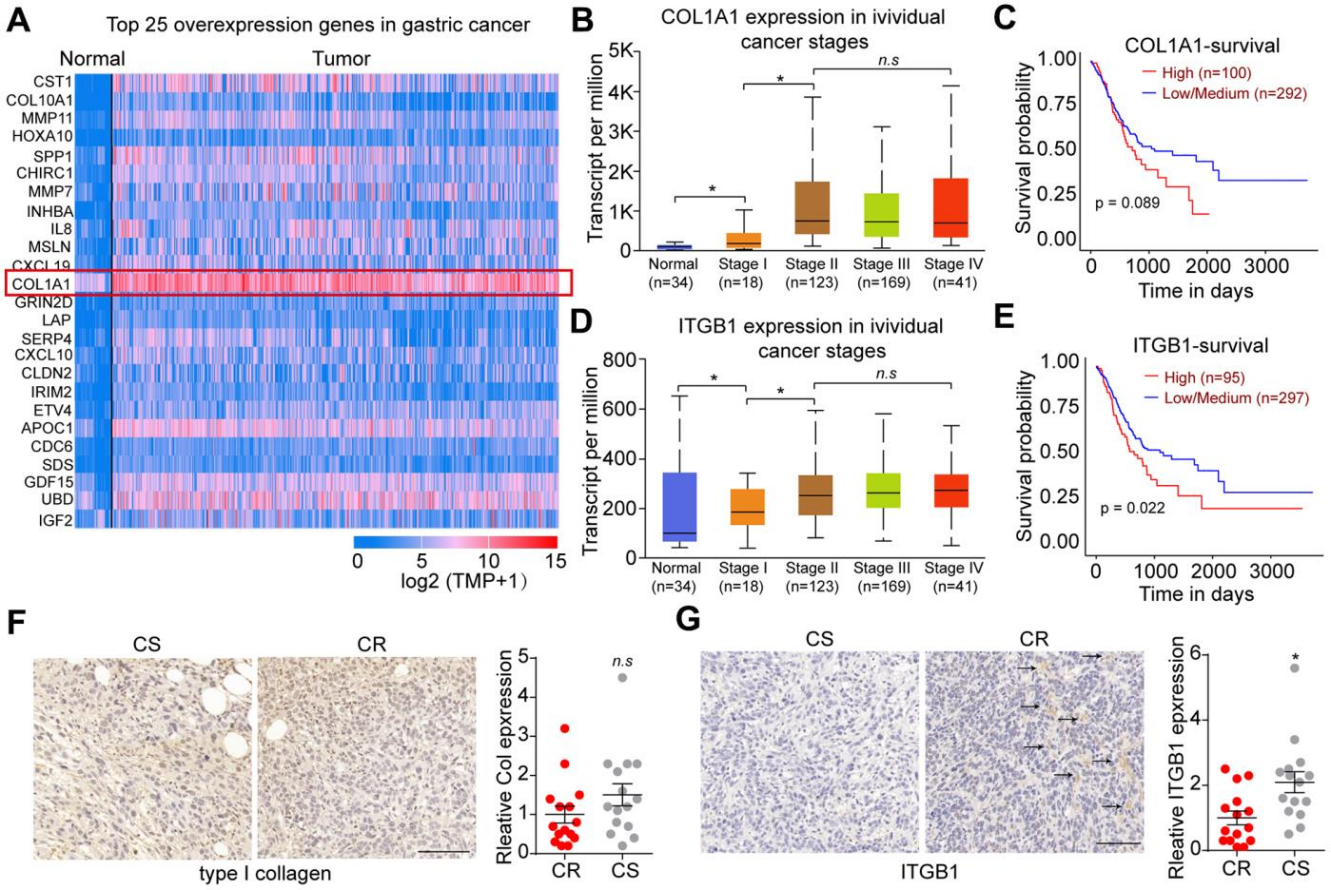
**Integrin β1 promoted gastric cancer progression.** (**A**) Top 25 overexpression genes in gastric tumor tissues comparing with normal tissues analyzed by TCGA database. (**B**) The relative expression of COL1A1 in normal tissues and gastric tumor tissues (stage I, II, III and IV) analyzed by TCGA database. (**C**) The overall survival of gastric cancer patients with low/high COL1A1 expression analyzed by TCGA database. (**D**) The relative expression of ITGB1 in normal tissues and gastric tumor tissues (stage I, II, III and IV) analyzed by TCGA database. (**E**) The overall survival of gastric cancer patients with low/high ITGB1 expression analyzed by TCGA database. (**F**) Relative expression of type I collagen in chemo-sensitive (CS) and chemo-resistant (CR) tumor tissues from gastric patients, which was examined by immunohistochemistry (n=15). The scale bar is 100 μm. (**G**) Relative expression of ITGB1 in chemo-sensitive (CS) and chemo-resistant (CR) tumor tissues from gastric patients, which was examined by immunohistochemistry (n=15). The scale bar is 100 μm. *Indicates P <0.05, n.s indicates no significant difference.

To explore the underlying mechanism of sustained tumor progression in gastric cancer, we first analyzed the TCGA database to comparing the top expression genes between gastric tumor tissues and normal tissues (Figure 1A). Notably, extremely high expression of COL1A1, which encodes type I collagen, was found in gastric cancer. To further confirm the pro-tumor effects of COL1A1 on gastric tumor progression, we used TCGA database to analyze the COL1A1 expression in gastric cancer patients with different stages and the overall survival. As shown in Figure 1B, elevated expression of COL1A1 was observed in gastric tumor tissues in high stage (stage II, III and IV) compared with normal tissues or tumor tissues in stage I (Figure 1B). However, no significant difference was found in overall survival of gastric cancer patients with high/low COL1A1 expression (Figure 1C), reminding us that COL1A1/type I collagen was not capable of regulating gastric tumor progression directly. Increasing evidence suggested that type I collagen could promote cancer stem cells proliferation and facilitate pro-survival signaling pathway activation through integrin receptors. Given the aberrant expression of COL1A1 in tumor tissues, we supposed that type I collagen might play a role through integrins signals in tumor development. ITGB1, belonging to the integrin family, has been demonstrated to bind to type I collagen and mediate the extracellular signals transduction [13, 14]. Intriguingly, using TCGA database analysis, elevated expression level of ITGB1 was found in gastric cancer tissues compared to normal tissues. Meanwhile, gastric cancer patients in high stage (stage II, III and IV) exhibited higher ITGB1 expression compared with patients in stage I (Figure 1D). More importantly, poor overall survival was observed in gastric cancer patients with high ITGB1 expression (Figure 1E). Subsequently, we further assessed the expression of type I collagen and ITGB1 in those chemo-resistant gastric tumor tissues. Consistently, no significant difference was observed in type I collagen expression between chemo-resistant (CR) and chemo-sensitive (CS) tumor tissues from gastric cancer patients (Figure 1F). As for ITGB1, elevated expression of ITGB1 was found in CR tumor tissues compared to CS group (Figure 1G). Together, those results suggested elevated expression of type I collagen and the positive correlation between ITGB1 and gastric cancer development.

### Type I collagen mediated gastric cancer progression through ITGB1

**Figure 2:**
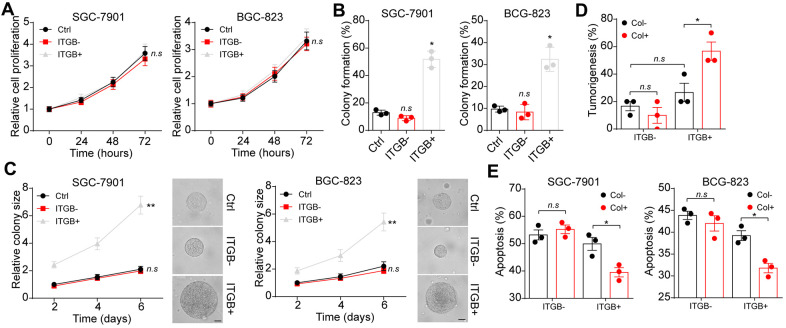
**Collagen mediated gastric cancer progression through integrin β1.** (**A**) The proliferation of unsorted or ITGB1-/+ SGC-7901/BGC-823 cells in 72 hours. (**B**) The 3D colony formation capability of unsorted or ITGB1-/+ SGC-7901/BGC-823 cells in 3D collagen gel. (**C**) The colony sizes of unsorted or ITGB1-/+ SGC-7901/BGC-823 cells in 3D collagen gel. The scale bar is 30 μm. (**D**) The ITGB1-/+ SGC-7901 cells were sorted and cultured in 3D collagen gel (6 days) or not. Then the tumorigenic capability of SGC-7901 in NOD-SCID mice were examined. (**E**) The ITGB1-/+ SGC-7901 cells were sorted and cultured 3D collagen gel (6 days) or not. Then tumor cells were treated with 5-FU (5 μg/ml) and the cell apoptosis was examined. *Indicates P <0.05, **Indicates P <0.01, n.s indicates no significant difference.

As ITGB1 correlated with the overall survival and tumor progression in gastric cancer patients, we thus questioned whether ITGB1 might regulate cells proliferation to promote gastric cancer development. To test this possibility, we sorted ITGB1 negative/positive gastric cancer cells (SGC-7901 and BGC-823) and examined the cells proliferation. However, no significant difference was observed in cell proliferation of ITGB1 positive/negative gastric cancer cells (Figure 2A). Given abundant expression of type I collagen in tumor tissues, we next seeded those sorted gastric cancer cells into 3D collagen gel. Intriguingly, ITGB1 positive SGC-7901 and BGC-823 cells revealed significantly enhanced capability of 3D colony formation (Figure 2B). More importantly, rapid colony growth was observed in ITGB1 positive cancer cells (Figure 2C), indicating that ITGB1 positive tumor cells possessed proliferative characteristics in the presence of collagen. Those results reminded us that type I collagen might mediate the tumor cells proliferation through the ITGB1 receptor. Meanwhile, the tumor progression induced by ITGB1 was dependent on the presence of type I collagen. To further confirm our hypothesis, we subcutaneously injected sorted ITGB+/- SGC-7901 cells into NOD-SCID mice. The 3D collagen cultured ITGB1+ tumor cells revealed strengthened tumorigenic capability compared to ITGB1 negative cells or dish cultured cells (Figure 2D). Subsequently, we further examined the effects of ITGB1 and collagen on drugs resistance. 5-FU is a nucleobase analogue that inhibits DNA synthesis to slow gastric tumor growth. Here, we sorted ITGB1+ and ITGB1- gastric cancer cells, which were seeded into 3D collagen gels or not. After 6 days, 5-FU was used to treat those tumor cells and the cytotoxicity of 5-FU was examined. Intriguingly, no significant drugs resistance was observed in ITGB1+ cells or collagen cultured ITGB1- tumor cells, whereas ITGB1+ cells cultured in 3D collagen gels revealed obvious 5-FU resistance (Figure 2E). This result indicated that collagen could promote drugs resistance of gastric cancer through ITGB1. Together, those results implicated that collagen could mediate the tumor progression and drugs resistance through the ITGB1 in gastric cancer.

### Type I collagen mediated the BCL9L expression through ITGB1

**Figure 3:**
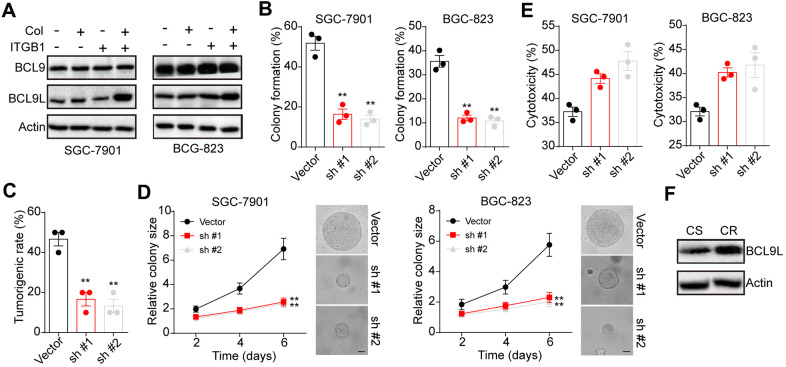
**BCL9L was up-regulated in 3D collagen cultured ITGB1+ gastric cancer.** (**A**) The ITGB1-/+ SGC-7901 cells were sorted and cultured in 3D collagen gel (6 days) or not. The expression of BCL9, BCL9L and β-actin was examined by western blotting. (**B**) The ITGB1+ SGC-7901 cells were sorted and cultured in 3D collagen gel (6 days). Then tumor cells were treated with BCL9L shRNA or vector and the 3D colony formation capability was examined. (**C**) The ITGB1+ SGC-7901 cells were sorted and cultured in 3D collagen gel (6 days). Then tumor cells were treated with BCL9L shRNA or vector and the tumorigenic capability was examined. (**D**) The colony sizes of tumor cells in (**C**). The scale bar is 30 μm. (**E**) The ITGB1+ SGC-7901 cells were sorted and cultured in 3D collagen gel (6 days). Then tumor cells were treated with BCL9L shRNA or vector. Then tumor cells were treated with 5-FU (5 μg/ml) and the cell apoptosis was examined. (**F**) Western blotting of BCL9L and β-actin in chemo-sensitive (CS) and chemo-resistant (CR) tissues from gastric patients. *Indicates P <0.05, ** Indicates P <0.01.

Next, we sought to investigate the underlying mechanism of ITGB1 associated gastric cancer progression and drugs resistance. BCL9 transcription coactivator has been reported to mediate pro-survival signaling pathways activation and correlated with lymphoma and leukemia development [15, 16]. Here, we further examined the expression of BCL9 in sorted ITGB+/- gastric cancer cells SGC-7901 and BGC-823(cultured in 3D collagen gel or not). No significant difference of BCL9 expression was observed in collagen cultured ITGB1 positive cells. However, upregulation of BCL9L, the paralog of BCL9 [17], was found in collagen cultured ITGB1 positive cells (Figure 3A). To further confirm the role of BCL9L in ITBG1 induced tumor progression, we silenced BCL9L in SGC-7901 and BGC-823 cells. As a result, blockade of BCL9L suppressed the 3D colony formation (Figure 3B) and tumorigenic capability (Figure 3C) of ITGB1 positive cells in 3D collagen gels. The colony growth of ITGB1 positive cells in 3D collagen gels was suppressed by BCL9L silencing (Figure 3D). Meanwhile, overexpression of BLC9L efficiently strengthened the colony formation capability of ITGB negative BGC-7901 and BGC-823 cells (Supplementary Figure 1A, 1B). Next, we further examined the association between BCL9L and drugs resistance in gastric cancer. Silencing BCL9L efficiently reversed the 5-FU resistance in collagen cultured ITGB1 positive cells (Figure 3E). Consistently, elevated expression of BCL9L was observed in tumor tissues from CR patients (Figure 3F). Together, those results suggested that BCL9L is in part relevant to the tumorigenic potential conferred by ITGB1.

### BCL9L induced activation of β-catenin/BCL2 signal pathway to regulate gastric cancer progression

**Figure 4:**
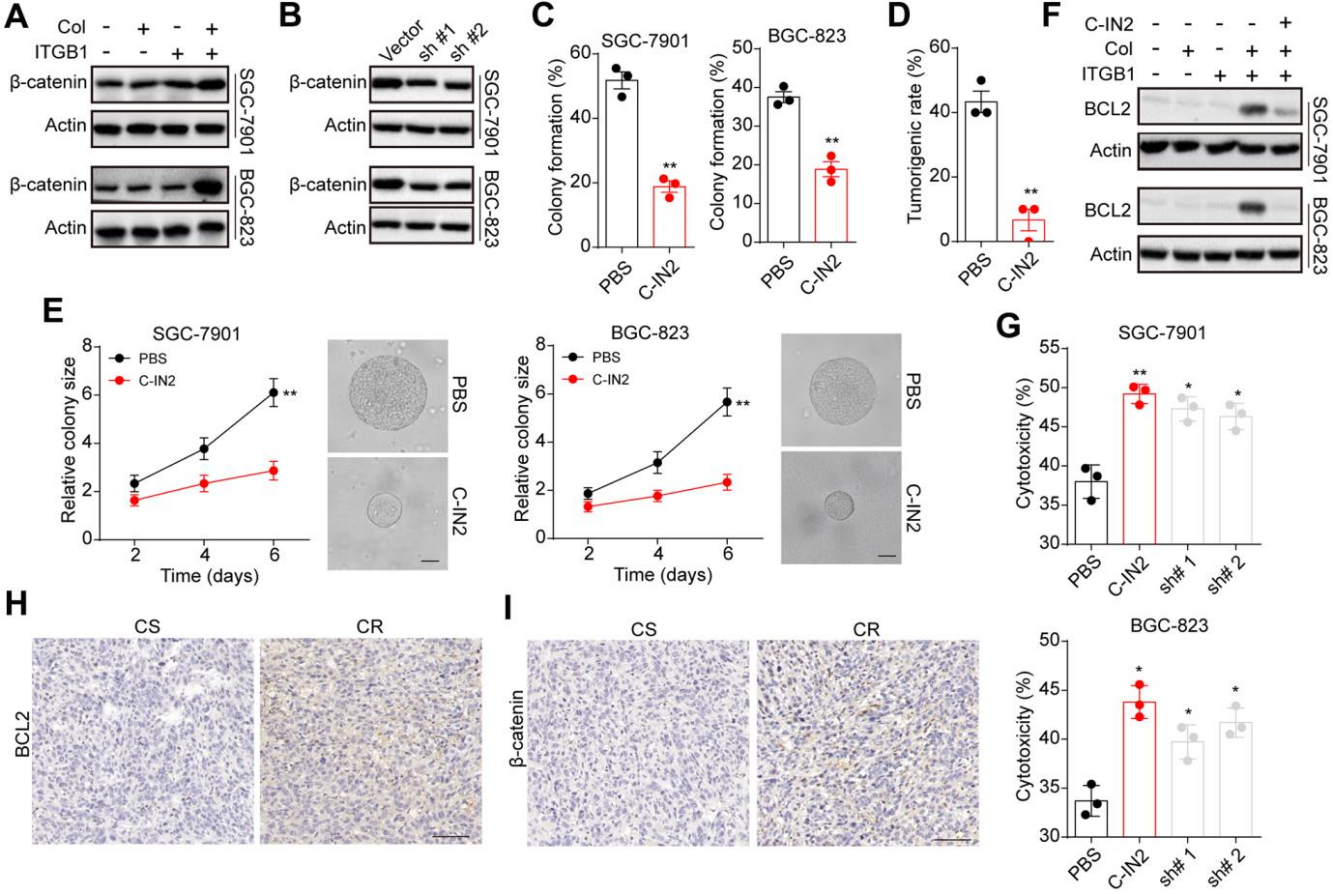
**BCL9L mediated gastric progression through downstream β-catenin and BCL2.** (**A**) ITGB1-/+ SGC-7901/BGC-823 cells were sorted and cultured with 3D collagen gel (6 days) or not. The expression of β-catenin and β-actin was examined by western blotting. (**B**) ITGB1+ SGC-7901/BGC-823 cells were cultured in 3D collagen gels (6 days) and treated with BCL9L shRNA or vector. Then the expression of β-catenin and β-actin was examined by western blotting. (**C**) ITGB1+ SGC-7901/BGC-823 cells were cultured in 3D collagen gels (6 days) and treated with PBS or C-IN2 (5 μM). Then the 3D colony formation capability was examined. (**D**) ITGB1+ SGC-7901/BGC-823 cells were cultured in 3D collagen gels (6 days) and treated with PBS or C-IN2 (5 μM). Then the 3D colony formation capability was examined. Then the tumorigenic capability was examined in NOD-SCID mice. (**E**) ITGB1+ SGC-7901/BGC-823 cells were cultured in 3D collagen gels (6 days) and treated with PBS or C-IN2 (5 μM). Then the colony sizes were examined. The scale bar is 30 μm. (**F**) ITGB1-/+ SGC-7901/BGC-823 cells were cultured in 3D collagen gel (6 days) and treated with PBS or C-IN2 (5 μM). Then the expression of BCL2 and β-actin was examined by western blotting. (**G**) ITGB1+ SGC-7901/BGC-823 cells were cultured in 3D collagen gel (6 days) and treated with PBS, C-IN2 (5 μM) or BCL2 shRNA. Then tumor cells were treated with 5-FU (5 μg/ml) and the apoptosis was examined. (**H**) Immunohistochemical staining of β-catenin in chemo-sensitive (CS) and chemo-resistant (CR) tissues from gastric patients. The scale bar is 100 μm. (**I**) immunohistochemical staining of BCL2 in chemo-sensitive (CS) and chemo-resistant (CR) tissues from gastric patients. The scale bar is 100 μm. * Indicates P <0.05, ** Indicates P <0.01.

Since Wnt/β-catenin served as the downstream signal of BCL9 [18, 19], we supposed that ITGB1 might upregulate BCL9L to mediate the activation of pro-survival β-catenin signals in gastric cancer. Here, we found enhanced expression of β-catenin in collagen cultured ITGB1 positive cells (Figure 4A). More importantly, suppression of BCL9L by sh RNA also suppressed the up-regulation of β-catenin in collagen cultured ITGB1 positive cells (Figure 4B), indicating that BCL9L mediated the activation of β-catenin signals in gastric cancer. Next, we used a β-catenin inhibitor β-catenin-IN2 (C-IN2) (Ann Marie Bode, et al. Inhibitors of beta-catenin in treatment of colorectal cancer. Patent US20150374662A1.) to treated collagen cultured ITGB1 positive cells. As a result, β-catenin inhibition obviously suppressed the 3D colony formation (Figure 4C) and tumorigenic rate (Figure 4D) of collagen cultured ITGB1 positive cells. The colony growth was inhibited by C-IN2 as well (Figure 4E). Subsequently, we examined the expression of anti-apoptotic protein BCL2, which have been reported to be up-regulated by β-catenin signals in tumor cells [20]. We found upregulation of BCL2 in collagen cultured ITGB1 positive cells, whereas suppression of β-catenin by C-IN2 suppressed BCL2 upregulation (Figure 4F). Subsequently, we suppressed β-catenin and BCL2 expression in collagen cultured ITGB1 positive cells, and then examined the cytotoxicity of 5-FU to gastric cancer cells. Consistently, suppression of β-catenin and BCL2 efficiently reversed the drugs resistance of collagen cultured ITGB1 positive cells (Figure 4G). And overexpression of BCL2 suppressed the effects of C-IN2 and strengthened the drugs resistance of collagen cultured ITGB1 positive cells (Supplementary Figure 1C, 1D). To further confirm the role of β-catenin and BCL2 in gastric resistance development, we examined the expression of β-catenin and BCL2 in tumor tissues from patients. Consistently, tumor tissues from CR gastric cancer patients revealed higher expression of BCL2 (Figure 4H) and β-catenin (Figure 4I). Those results suggested that BCL9L mediated activation of β-catenin/BCL2 signal pathway to regulate gastric cancer progression.

### Suppression of β-catenin signals improved outcome of chemotherapy in gastric cancer

**Figure 5:**
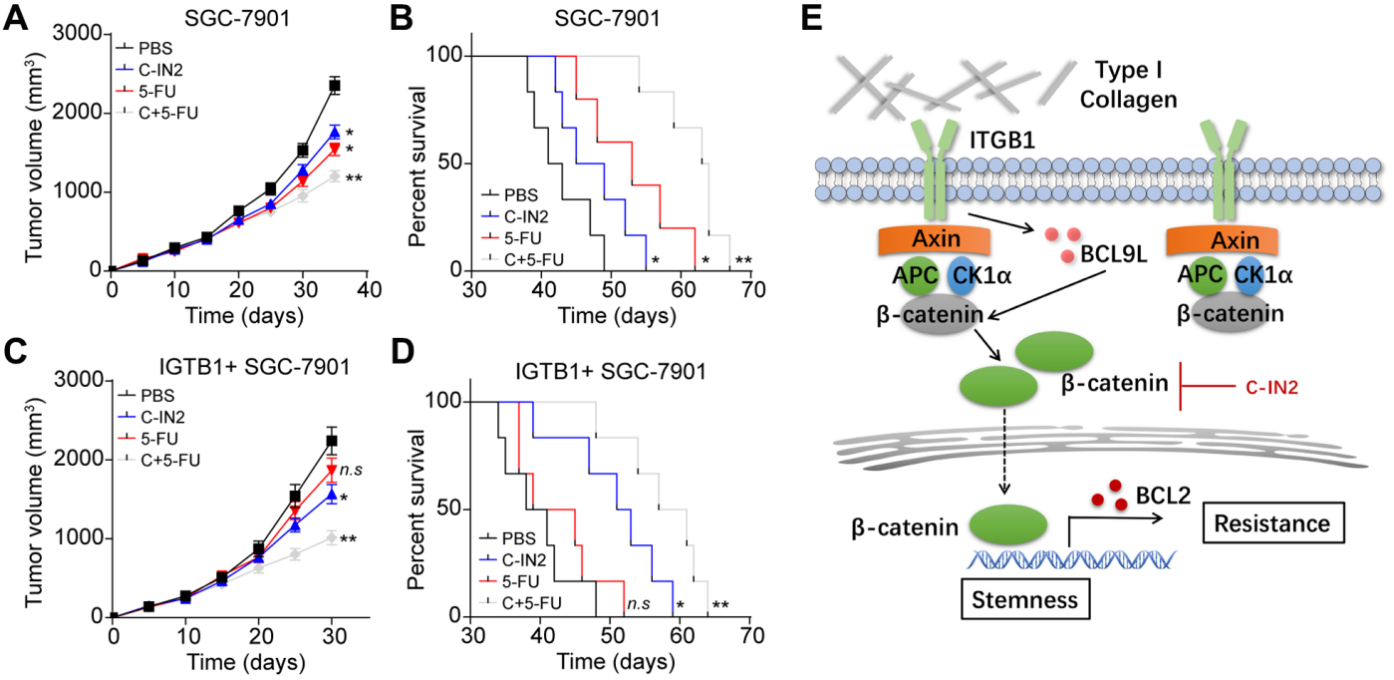
**Blockade of β-catenin signals improved outcome of chemotherapy.** (**A**) Tumor volume of SGC-7901 bearing mice treated with PBS, C-IN2, 5-FU and C-IN2 combined with 5-FU. (**B**) Overall survival of SGC-7901 bearing mice treated with PBS, C-IN2, 5-FU and C-IN2 combined with 5-FU. (**C**) Tumor volume of ITGB1+ SGC-7901 bearing mice treated with PBS, C-IN2, 5-FU and C-IN2 combined with 5-FU. (**D**) Overall survival of ITGB1+ SGC-7901 bearing mice treated with PBS, C-IN2, 5-FU and C-IN2 combined with 5-FU. (**E**) Schematic diagram of ITGB1 induced tumor progression in gastric cancer. * Indicates P <0.05, ** indicates P <0.01, n.s indicates no significant difference.

Our previous results have provided evidence that activation of β-catenin signals induced by collagen/ITGB1 could facilitate the chemotherapy resistance in gastric cancer. To improve the outcome of chemotherapeutic agents and suppress gastric cancer progression, it might be feasible to block β-catenin signals to improve the anticancer effects of chemotherapy. Here, we employed C-IN2 combing chemotherapeutic 5-FU to treat subcutaneous gastric tumor bearing mice model. Gastric tumor bearing mice models were constructed by subcutaneously injecting SGC-7901 cells into NOD-SCID mice. The tumor bearing mice were grouped randomly and received PBS, 5-FU monotherapy or combining with C-IN2 on day 14. After treatment of 2 weeks, we found that 5-FU or C-IN2 monotherapy could suppress the tumor growth slightly, whereas combination group revealed significant tumor suppressive effects (Figure 5A). Additionally, the mice in combination group exhibited obvious prolonged survival time compared to PBS or monotherapy groups (Figure 5B and Table 1), indicating that suppression of β-catenin signals strengthened the anticancer effects of chemotherapy. To further explore the anticancer effects of C-IN2 in ITGB1 positive gastric tumor tissues, we established subcutaneous gastric tumor bearing mice model by injecting 3D collagen cultured ITGB1 positive SGC-7901 cells into NOD-SCID mice, then mice were treated mice with PBS, 5-FU monotherapy or combining with C-IN2. Intriguingly, 5-FU monotherapy could not suppress the tumor growth or prolonged survival time of tumor bearing mice, which might be due to the drugs resistance induced by ITGB1. However, 5-FU combing C-IN2 revealed obvious anticancer effects (Figure 5C, 5D and Table 2). These results reminded that blockade of β-catenin signals to combine with chemotherapy could efficiently reverse the drugs resistance induced by ITGB1 and improved the anticancer effects, providing an innovative approach for gastric cancer therapy.

**Table 1:** The median survival and maximum survival time of SGC-7901 bearing mice treated with PBS, C-IN2, 5-FU and C-IN2 combined with 5-FU.

**Group**	**Median survival (days)**	**Maximum survival (days)**
PBS	41	49
c-IN2	45	55
5-FU	50	62
C+5FU	63	67

The median survival and maximum survival time of SGC-7901 bearing mice treated with PBS, C-IN2, 5-FU and C-IN2 combined with 5-FU.

**Table 2:** The median survival and maximum survival time of ITGB1+ SGC-7901 bearing mice treated with PBS, C-IN2, 5-FU and C-IN2 combined with 5-FU.

**Group**	**Median survival (days)**	**Maximum survival (days)**
PBS	38	48
c-IN2	51	59
5-FU	39	52
C+5FU	57	64

The median survival and maximum survival time of ITGB1+ SGC-7901 bearing mice treated with PBS, C-IN2, 5-FU and C-IN2 combined with 5-FU.

## DISCUSSION

The mechanism of extracellular matrix induced tumor progression is still poorly understood. In our study, we provided evidence that type I collagen served as fundamental component in extracellular matrix to facilitate the tumor progression through an ITGB1 dependent manner. ITGB1 positive gastric cancer cells possessed strengthened capability of colony formation and proliferative characteristics in the presence of type I collagen. More importantly, we demonstrated that type I collagen could upregulate the expression of BCL9L through ITGB1, eventually resulting in the activation β-catenin signaling pathway. Subsequently, the intranuclear β-catenin further up-regulated anti-apoptosis protein BCL2, leading to the chemotherapy resistance (Figure 5E). Here, we combined β-catenin inhibitor C-IN2 with chemotherapeutic agents to treat subcutaneous SGC-7901 tumor bearing mice. C-IN2 treatment efficiently strengthened the anticancer effects of 5-FU and prolonged the survival time of tumor bearing mice, which described novel strategy for gastric cancer.

Among the components with significant correlation to tumor progression, collagen family have been emerging as crucial participant in regulating tumor progression [21]. A bulk of collagen associated genes have previously been proved to be upregulated and participated in the process of tumor development, such as COL6A3 in pancreatic cancer [22]. Compelling findings have implicated that the presence of type I collagen and related signaling pathway are tightly associated with chemo-resistance and tumor metastasis [14, 23]. Increasing studies also suggested that the 3D collagen culture based on type I collagen could facilitate breast cancer cells stemness and invasion *in vitro* [24]. However, the correlation analysis based on TCGA database or clinical samples implicated that the expression of type I collagen is prone to be not positive correlated to tumor progression in several tumor types. Here, we observed abundant type I collagen distribution in gastric cancer tissues in all tumor stages. No significant difference was observed in the overall survival between patients with high/low type I collagen expression. However, we found the presence of type I collagen could promote ITGB1 positive gastric cancer cells colony formation and growth, thereby resulting in the tumor progression. More importantly, the expression of ITGB1 revealed a high correlation with the overall survival of gastric cancer patients. Our results further disclosed the role of type I collagen in tumor progression, which is dependent on collagen binding integrin receptor ITGB1. Given the abundant expression of collagen in most tumor types, it might be befitting to focus on the expression of collagen binding integrin receptors for tumor diagnosis or prediction.

Increasing evidence have suggested that collagen could mediate activation of specific pro-survival signaling pathways in diverse tumor cells. Type VI collagen was recently demonstrated to inhibit apoptosis through suppressing the expression of Bax [25]. Type I collagen could mediate the prostate cancer invasion through RhoC GTPase and integrin α2β1 signals [26]. Moreover, some pro-survival signals, such as FAK [27], AKT [28] and STAT3 [29, 30], are reported to be upregulated by integrins signals in cancer cells. Our study further demonstrated that type I collagen could mediate the activation of ITGB1/BCL9L/β-catenin signaling pathway, leading to the tumor progression and drugs resistance. Previous reports have implicated the role of BCL9 in tumor development [31, 32] and our study further disclosed the pro-tumor effects of BCL9 paralog BCL9L, which could promote the β-catenin signals activation in gastric cancer. Given the crucial role of β-catenin in chemo-resistance development, we then suppressed the β-catenin signals by C-IN2 to improve outcome of chemotherapy. Notably, C-IN2 treated groups revealed strengthened anticancer effects and efficiently reversed the drugs resistance induced by ITGB1 and collagen. More importantly, no significant weight loss or side effects were observed in C-IN2 treated mice, suggesting the potential application of β-catenin inhibitors in gastric cancer treatment. However, the further application of integrin inhibitors in gastric needed to be validated in larger cohort of gastric cancer patients. And the systemic toxicity evaluation of combination therapy remained to be further assessed. Meanwhile, knockdown of β-catenin by Crisp/Cas9 or shRNA to explore the role of β-catenin in gastric cancer development might further demonstrated our hypothesis. Our study indicated the elevated expression of ITGB1 in gastric cancer and its’ association with collagen, which might be benefit from targeted therapies in further clinical trials.

In conclusion, we showed that type I collagen could mediate the tumor progression and chemotherapy though an ITGB1 based manner, which was dependent on BCL9L/β-catenin/BCL2 signaling pathway. Blockade of β-catenin strengthened the anticancer of chemotherapy, describing an innovative approach for gastric cancer treatment.

## MATERIALS AND METHODS

### Cell lines and reagents

Human gastric cancer cells SGC7901 and BGC-823 were purchased from the American Type Culture Collection (Maryland, USA) and maintained in 1640 complete medium (Gibco, CA, USA) supplemented with 10% fetal calf serum (Gibco, CA, USA) at 37° C in 5% CO2 atmosphere. β-catenin inhibitor β-catenin-IN2 (C-IN2) was purchased from MedChemExpress (N.J, USA). 5-FU was purchased from Sangon (Shanghai, China). Type I collagen and collagenase were purchased from Thermo (MA, USA). Other reagents were purchased from Solarbio (Beijing, China) and of HPLC standard.

### Patients’ tumor tissues collection

The tumor tissues of gastric cancer patients were obtained from the Fourth Hospital of Hebei Medical University. Samples were collected and sent to the laboratory within 2 hours. According to the response to chemotherapy in our follow-up study, the tumor tissues were divided into chemo-resistant (CR) and chemo-sensitive (CS) groups. All tumor samples were in stage T2~T3 and the follow-op study was performed after surgical operation. All samples collection and processing were carried out respecting the Declaration of Helsinki. All patients signed informed consent prior to tumor tissues collection treatment, including allowing their data to be used for further research. All subjects gave written informed consent. Ethical approval was obtained from the Committee of the Fourth Hospital of Hebei Medical University.

### 3D collagen gel culture

3D collagen gel culture was performed according previous report [33]. Briefly, type I collagen was diluted to 0.6 mg/ml with culture medium. 5 × 10^4^ sorted tumor cells were added into the collagen solution and mixed thoroughly. Next, 25 μl 10 × PBS and 20 μl NaOH (1 M) solution were added into the solution, and 295 μl of the mixture was seeded into 24-well plate. After 2 hours of 37° C incubation, the solid clotty collagen (containing tumor cells) was removed into RPMI 1640 complete medium supplemented with 10% fetal calf serum for cells culture. After 4~6 days, the cells were digested by type I collagenase and trypsin for further analysis.

### Cell proliferation and 3D colony formation analysis

The cell proliferation of SGC-7901 and BGC-823 was performed using a MTT analysis kit (Solarbio, Beijing, China). Briefly, 3000 sorted tumor cells were seeded into the 96-well plates with 200 μl 1640 complete medium supplemented with 10% fetal calf serum. After 24, 48 and 72 hours, 5 μl MMT solution was added into the 96-well plated. After 2 hours of incubation, the culture medium was removed and 100 μl DMSO was added into the 96-wells. Absorbance was measured at 570 nm on a microplate reader (Bio-Rad, MA, USA). For the 3D colony formation assay, sorted tumor cells (~200 cells/well) were seeded in the 3D collagen gel. After 4 days, the cell colonies were imaged and counted. The colony sizes were calculated by image J 2.3 software. 50 colonies were pictured and the sizes were calculated in each group. Each experiment was performed three times, independently.

### Gene interference

All constructs for functional analyses were designed according to the lentiviral plasmid pHR-SIN-CSGW-ΔNot, which was generated by partial NotI-digest and subsequent fill-in reaction of one of the 2 NotI sites originally present in the primary lentiviral plasmid pHR-SIN-CSGW10 (Hanbio, Beijing, China). Complementary shRNA oligonucleotides directed against either BCL9L or BCL2 were synthesized by Hanbio (Beijing, China). Each experiment was performed three times, independently.

### Cells apoptosis analysis

Cells apoptosis was analyzed using the FITC-Annexin V and PE-PI apoptosis detection kit (BD, NJ, USA). Briefly, tumor cells were collected and stained with FITC-Annexin V and PE-PI viability staining solution for 15 min. Subsequently, apoptosis was detected by flow cytometry on flow cytometer (Accuri^®^C6, Becton Dickinson, NJ, USA). Each experiment was performed three times, independently.

### Flow cytometry

SGC-7901 and BGC-823 cells were were harvested, washed twice with cold PBS buffer and stained with a ITGB1 antibody (eBioscience, MA, USA) for 30 minutes at room temperature. The samples were then washed and sorted with flow cytometer (Accuri^®^C6, Becton Dickinson, NJ, USA). Each experiment was performed three times, independently.

### Immunohistochemical staining

Gastric tissues dissected from patients were fixed in 4% paraformaldehyde for 48 hours and sectioned at a thickness of 4 μm. The sections were then deparaffinized and rehydrated in alcohol and water. Antigen retrieval was performed in sodium citrate buffer for five minutes at 100° C. Hydrogen peroxide (0.3%) was used to block peroxidase. Then, the sections were incubated with anti-collagen (1:200, Abcam, Cambridge, UK), anti-ITGB1 (1:200, Abcam, Cambridge, UK), anti-BCL2 (1:200, Abcam, Cambridge, UK) or anti-β-catenin (1:200, Abcam, Cambridge, UK) primary antibodies at 4° C overnight. After being washed with PBS, the samples were incubated with goat anti-rabbit secondary antibodies (HRP, 1:1000, Abcam, Cambridge, UK). Images were obtained using microscope (DM4 M, Leica, Germany) and the relative intensity of proteins was calculated by Image-Pro Plus 6.0 software. 15 tumor samples were calculated in each group (CS and CR groups). 20 fields were pictured for protein intensity analysis in each sample and at least 100 tumor cells were pictured in each field. Using the 30 samples, each experiment was performed at least three times, independently.

### Western blotting

Radioimmunoprecipitation assay buffer (Beyotime, Beijing, China) containing protease inhibitors (Beyotime, Beijing, China) was used to lyse the treated cells. 20 μg protein samples were separated via sodium dodecyl sulfate polyacrylamide gel electrophoresis and transferred onto nitrocellulose membranes. The following primary antibodies were used: anti-BCL9L (1:500, Abcam, Cambridge, UK), anti-BCL9 (1:500, Abcam, Cambridge, UK), anti-β-actin (1:1000, Abcam, Cambridge, UK) and anti-β-catenin (1:5000, Abcam, Cambridge, UK). Proteins were detected using a chemiluminescence kit (Beyotime, Beijing, China). The expression of β-actin was used as an internal control. Each experiment was performed three times, independently.

### Animal protocols

Female NOD-SCID mice (6–8 weeks old) were purchased from Huafukang (Beijing, China). All mice were housed in a specific pathogen-free facility. All animal protocols in our experiments were approved by the Institutional Animal Care and Use Committee of the Fourth Hospital of Hebei Medical University, Protocol ID: EMC20121128c. All applicable institutional and governmental regulations concerning the ethical use of animals were followed. For tumorigenic analysis, 10^5^ SGC-7901 cells were digested and resuspended in PBS and subcutaneously injected into the NOD-SCID mice. After 25 days, the tumorigenic rate of SGC-7901 cells in NOD-SCID mice was analyzed. 10 mice were calculated in each group.

For tumor suppression analysis, 10^6^ SGC-7901 or ITGB1+ SGC-7901 cells were digested and resuspended in PBS and subcutaneously injected into the NOD-SCID mice. On days 14, mice were treated with PBS, C-IN2 (5 mg/kg, 200 μl per mouse), 5-FU (10 mg/kg, 200 μl per mouse) and C-IN2 (5 mg/kg, 200 μl per mouse) combined with 5-FU (5 mg/kg, 200 μl per mouse) by tail vein injection twice a week. In combination group, 5-FU was injected into tumor bearing mice after 24 hours of C-IN2 treatment. The tumor volume was calculated as follows: length × width^2^ × 0.5. Survival was recorded on a daily basis. All animal experiments were monitored by the Fourth Hospital of Hebei Medical University. 6 mice were calculated in tumor volume and survival analysis in each group. Each experiment was performed three times, independently.

### Statistical analysis

Each experiment was performed at least three times, independently. Results were presented as the mean ± SEM. All statistical significance between groups was calculated by Student’s t test for two groups (Figures 1F, 1G, 4C–4E), and all statistical significance between groups was calculated by one-way ANOVA for more than two groups. The survival rates were determined by Kaplan–Meier survival analysis (*p < 0.05; **p < 0.01; ***p < 0.001; ns, no significant difference).

## Supplementary Material

Supplementary Figure 1
