# Supplementary material for: Type I collagen promotes tumor progression of integrin β1 positive gastric cancer through a BCL9L/β-catenin signaling pathway
Source: Aging (Albany NY). 2021 Jul 28;13(14):19064–76. doi: 10.18632/aging.203355 (PMC8351671; doi:10.18632/aging.203355)
Supplement: Supplementary Figure 1 [file aging-13-203355-s001.pdf]

SUPPLEMENTARY FIGURE

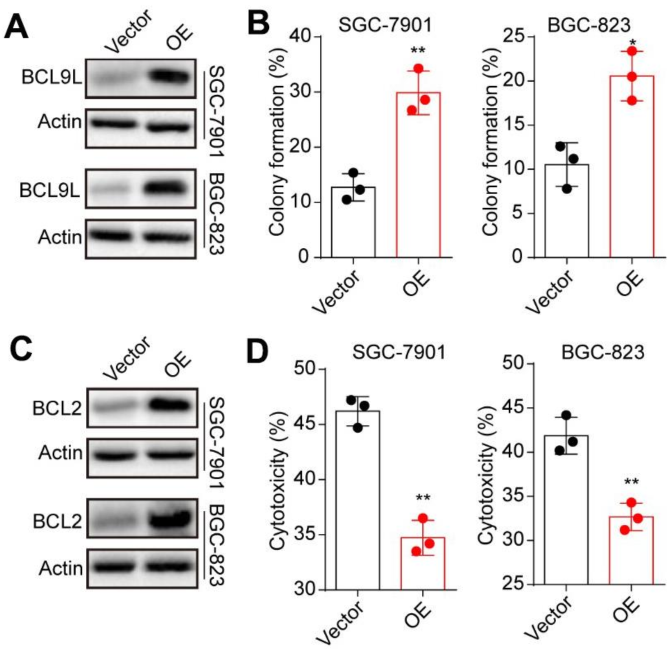

Supplementary Figure 1. (**A**) Western blotting of BCL9L in SGC-7901/BGC-823 (Vector) and BCL9L overexpression SGC-7901/BGC-823 cells (OE). (**B**) ITGB1- SGC-7901/BGC-823 (Vector) and BCL9L overexpression SGC-7901/BGC-823 cells (OE) were sorted and seeded in 3D collagen gels. The colony formation rates were calculated on day3. (**C**) Western blotting of BCL2 in SGC-7901/BGC-823 (Vector) and BCL2 overexpression SGC-7901/BGC-823 cells (OE). (**D**) The ITGB1+ SGC-7901/BGC-823 (Vector) and BCL2 overexpression SGC-7901/BGC-823 cells (OE) cells were sorted and cultured in 3D collagen gel (6 days). Then tumor cells were treated with 5-FU (5 µg/ml) combing C-IN2 (5 µM) and the cell apoptosis was examined. * Indicates $P < 0.05$, ** indicates $P < 0.01$.
